# Intensity of care in cancer patients in the last year of life: a retrospective data linkage study

**DOI:** 10.1038/s41416-022-01828-0

**Published:** 2022-05-11

**Authors:** Xhyljeta Luta, Katharina Diernberger, Joanna Bowden, Joanne Droney, Peter Hall, Joachim Marti

**Affiliations:** 1 Centre for Primary Care and Public Health (Unisanté), University of Lausanne, Lausanne, Switzerland; 2 Institute of Global Health Innovation, Department of Surgery and Cancer, Imperial College London, London, UK; 3 Lausanne University Hospital (CHUV), Lausanne, Switzerland; 4 University of Edinburgh, Edinburgh Clinical Trials Unit, Usher Institute, Edinburgh, UK; 5 Edinburgh Cancer Research Centre, University of Edinburgh, Edinburgh, UK; 6 NHS Fife, Scotland, UK; 7 University of St Andrews, Scotland, UK; 8 The Royal Marsden NHS Foundation Trust, London, UK

**Keywords:** Cancer, Cancer

## Abstract

**Background:**

Delivering high-quality palliative and end-of-life care for cancer patients poses major challenges for health services. We examine the intensity of cancer care in England in the last year of life.

**Methods:**

We included cancer decedents aged 65+ who died between January 1, 2010 and December 31, 2017. We analysed healthcare utilisation and costs in the last 12 months of life including hospital-based activities and primary care.

**Results:**

Healthcare utilisation and costs increased sharply in the last month of life. Hospital costs were the largest cost elements and decreased with age (0.78, 95% CI: 0.73–0.72, *p* < 0.005 for age group 90+ compared to age 65–69 and increased substantially with comorbidity burden (2.2, 95% CI: 2.09–2.26, *p* < 0.005 for those with 7+ comorbidities compared to those with 1–3 comorbidities). The costs were highest for haematological cancers (1.45, 95% CI: 1.38–1.52, *p* < 0.005) and those living in the London region (1.10, 95% CI: 1.02–1.19, *p* < 0.005).

**Conclusions:**

Healthcare in the last year of life for advanced cancer patients is costly and offers unclear value to patients and the healthcare system. Further research is needed to understand distinct cancer populations’ pathways and experiences before recommendations can be made about the most appropriate models of care.

## Background

The rising costs of diagnosing and treating cancer in its early stages, as well as delivering high-quality palliative and end-of-life care for people with cancer pose major challenges for health services in England and other high-income countries [1]. A surge in novel cancer treatments over the last decade has undoubtedly improved outcomes for many but has come with important additional provider costs [2]. Cancer is the second most common cause of death in England accounting for 28% of deaths (>130,000 people in England died of cancer in 2019). Furthermore, it is predicted that by 2040 a quarter of people aged 65 and above will have cancer [3]. Thus, with rising demands for the whole spectrum of cancer care from diagnosis, through treatment, to end-of-life care, health economic appraisal of the value of different models of care and treatments is essential.

It has been long recognised that people with cancer who are nearing the end-of-life are at risk of over medicalisation [4, 5]. More recent research has shown that hospital-based clinical interventions at the end-of-life can negatively impact both quality of life and care satisfaction [6]; furthermore, such interventions are often not in line with patients’ needs and preferences. A majority of deaths of people with cancer take place in hospitals, despite expressed preferences by many for end-of-life care at home [7]. A recent UK study [8], revealed that 58% of people with advanced haematological cancers died in hospital, despite 80% having previously identified the home as their preferred place of death.

In parallel with the potential negative impact on patients, highly medicalised end-of-life care significantly increases healthcare costs. Our own recently published data revealed that people dying of cancer were at higher risk of acute hospital admission towards the end-of-life, when compared with people dying of non-cancer conditions, and that this was associated with significantly higher costs. Healthcare use and costs were particularly high during the last 30 days of life, when hospital-based clinical interventions may offer limited benefit. The main drivers of resource use and costs were related to inpatient hospital care [9, 10]. Similar findings have been reported by other researchers [11] with Langton et al. estimating that 40% of costs in the last year of life were accumulated during the last month of life [12].

A systematic review examining resource utilisation towards the end-of-life for people with cancer revealed a range of factors that were associated with higher healthcare use and costs, including gender and ethnicity, comorbidity burden and rurality [12]. The majority of studies included in the review were from North America, with studies facilitated by the availability of large scale insurance and administrative datasets. The extent to which the observed patterns of healthcare use and the predictors of high healthcare use and costs are transferable to the UK is unknown.

The present research builds on our earlier, larger study examining healthcare utilisation and costs for decedents of all causes [9, 10]. Here, we focus on cancer patients and examine the intensity of cancer care in England in the last 12 months of life. We describe primary and secondary healthcare utilisation and associated costs by cancer types and other patient characteristics.

## Methods

### Study population

We used data from Clinical Practice Research Datalink (CPRD). CPRD collects routine data from primary care practices covering ~7% of the UK population [13]. Our study was limited to practices and patients based in England. The primary care dataset was linked to routinely collected secondary care information from Hospital Episode Statistics (HES) (hospitalisations, Accident & Emergency (A&E) contacts, intensive care unit (ICU) use and hospital outpatient setting) and Office for National Statistics (ONS) (death records). A detailed description of each dataset is reported in our previous study [10].

We studied decedents aged 65 years and older who died between January 1, 2010 and December 31, 2017 with a cancer diagnosis recorded in the 12 months before death that was a certified primary cause of death. Cancer diagnoses were identified from International Classification of Diseases version 10 (ICD-10) [14] codes within HES records [15]. Causes of death were extracted from death registrations held by the ONS.

The final sample included 26,077 individuals who we categorised, by cause of death, into one of ten main cancer groups based on prevalence in the dataset: lung, digestive organs (oesophagus, liver, stomach and other digestive organs other than pancreas), prostate, haematological (lymphomas and other haematological neoplasms), unspecified primary sites, colorectal, breast, urinary tract, pancreatic, female reproductive organs and other (less frequent) cancers. Decedents were characterised by a range of demographic variables including sociodemographic status, age, gender, region of residence, Index of Multiple Deprivation (IMD) [16] and clinical characteristics, including cancer type, comorbid conditions (identified from ICD-10 codes in HES records in the final 5 years of life) and comorbidity burden [17].

### Measures of the intensity of care

We examined healthcare utilisation and associated costs in the last 12 months of life for all decedents. We assessed the intensity of hospital inpatient care using the following indicators: number of hospital admissions, total length of hospital stays (in days), number of Accident and Emergency visits, intensive care unit (ICU) use including number of ICU admissions, and length of stay. For hospital outpatient care we calculated the mean number of outpatient appointments per patient in the last year of life. Primary care data included all clinical contacts, encompassing GP appointments, telephone assessments, home visits and out-of-hours consultations and prescriptions.

We examined healthcare costs in the last year of life based on the identified secondary care and primary care (including out-of-hours) contacts. The costing of hospital stays was based on payment tariffs assigned to each stay based on Healthcare Resource Group (HRG) code as reported in the National Schedules of Reference Costs [18]. Prescription costs were obtained from standard reference sources including the Personal Social Services Research Unit (PSSRU) [19]. Costs for outpatient appointments were derived from national schedules of reference costs, based on national average unit costs provided for each service (service code). A more detailed description of the costing methodology can be found in our previous publication [10].

In addition to the last year of life, we also looked at healthcare use in the last 90 days of life and last 30 days of life [20, 21], as timeframes when deteriorating health may have been more evident clinically, and when the benefit versus the burden of healthcare interventions may arguably have been less clear.

### Statistical analysis

We undertook descriptive statistical analysis to characterise the study population and their healthcare use. Outcome measures were described by means (SD) and frequencies (%). In addition, we conducted a subset analysis across cancer types: lung, digestive organs (oesophagus, liver, stomach and other digestive organs), prostate, haematological (lymphomas and other haematological neoplasms), unspecified primary sites, colorectal, breast, urinary tract, pancreatic, female reproductive organs and other cancers. We used generalised linear models (GLM) to analyse factors associated with costs including time to death, age, gender, comorbidity burden, cause of death, region of residence and IMD on healthcare use. We performed poison regression to explore more specific predictors for hospital admissions. For reference purposes only, we compared healthcare costs between patients who died of cancer and those who died of all other causes.

Data management and analysis were conducted using Stata version 15 (StataCorp, College Station, TX, USA). Code is available upon request.

## Results

### Cohort characteristics

The characteristics of the study population are shown in Table 1. The mean age at death was 80.9 years, 52.6% were men. Over 70% of cancer decedents had at least one or more comorbidities and the largest proportion (55.4%) had between 1 and 3 comorbidities. The most frequent cancers were lung (19%), digestive organs (including oesophagus, liver and stomach and other digestive organs (16%), prostate (8.4%), lymphomas and other haematological neoplasms (8.0%). The breakdown of demographics by cancer type is shown in Supplementary Appendix 1. The prevalence of almost all cancers (digestive, prostate, haematological, colorectal, pancreatic, breast, female reproductive organs and urinary tract) was higher in the most deprived group of decedents compared to other IMD groups.

**Table 1:** Patients’ characteristics, 2010–2017.

Characteristics	*N*	%
Gender		
Female	13,719	52.61
Male	12,358	47.4
Age		
65–69	1613	6.2
70–79	9893	37.9
80–89	10,767	47.3
90+	3804	14.6
Cancer site		
Lung	5166	19.8
Digestive organs^a^	4360	16.7
Prostate	2093	8.4
Hematological^b^	2093	8.0
Unspecified primary sites	1740	6.7
Colorectal	1734	6.6
Breast	1663	6.4
Urinary tract	1625	6.2
Pancreatic	1383	5.3
Female reproductive organs	1129	4.3
Other	2987	11.4
Charlson Comorbidity Index		
0	6909	26.5
1–3	14,455	55.4
4–6	2261	8.7
7+	2452	9.4
IMD^c^		343
1st (most deprived)	6039	23.2
2nd	5806	22.3
3rd	5740	22.0
4th	4725	18.1
5th (least deprived)	3767	14.4
Region		
South East Coast	5298	20.3
South Central	4224	16.2
North West	4037	15.5
West Midlands	3359	12.8
South West	3107	11.9
London	2882	11.0
East of England	2172	8.3
Yorkshire & The Humber	578	2.2
North East	420	1.6

^a^Digestive organs included oesophagus, liver, stomach and other digestive organs.

^b^Lymphomas and other haematological neoplasms including hodgin lymphoma, follicular and non-follicular lymphoma, mature T/NK-cell lymphomas, lymphoma T/NK-cell–cell lymphomas, malignant immunoproliferative diseases, multiple myleoma and malignant plasma cell neoplasms, lymphoid leukaemia, myeloid leukaemia, monocytic leukaemia and other leukaemias.

^c^*IMD* Index of Multiple Deprivation.

**Fig. 1:**
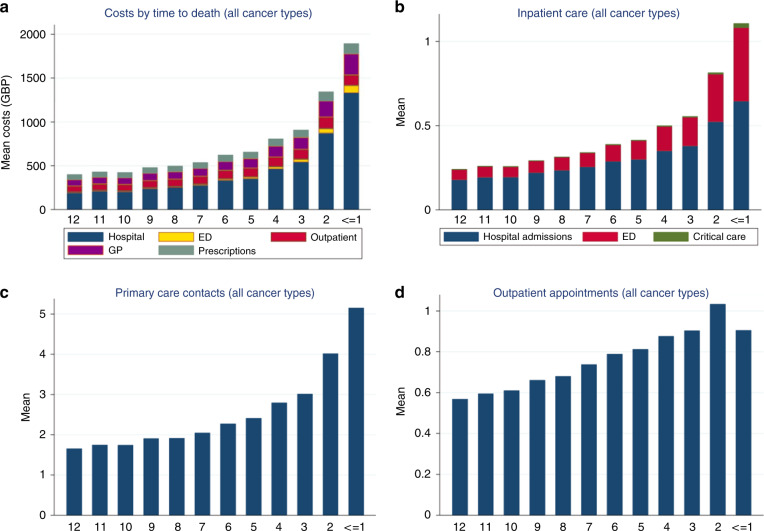
Healthcare use and costs in the last 12 months of life across all cancer types. Costs by time to death (**a**); inpatient care (**b**); primary care contacts (**c**); and outpatient appointments (**d**).

**Fig. 2:**
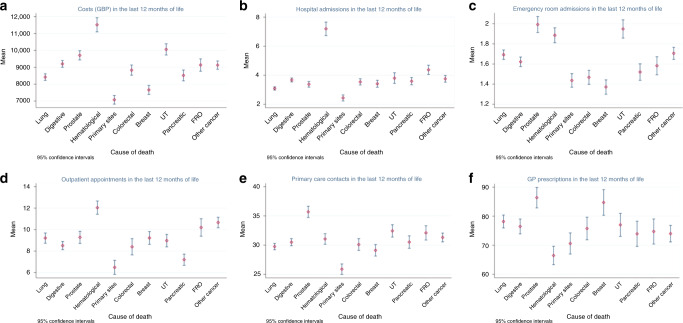
Healthcare use and costs in the last 12 months of life by cancer type. Costs (GBP) by time to death (**a**); hospital admissions (**b**); emergency room admissions (**c**); outpatient appointments (**d**); primary care contacts (**e**); and General Practitioner (GP) prescriptions (**f**).

**Fig. 3:**
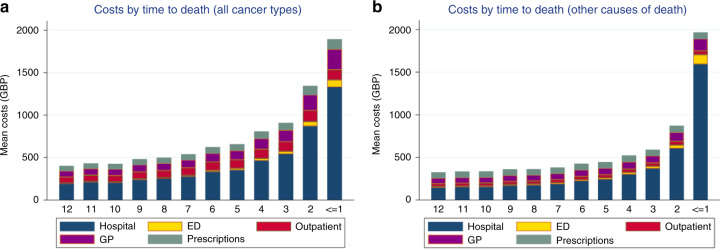
Healthcare costs in the last 12 months of life: cancer and other causes of death. Costs by time to death across all cancer types (**a**); and costs by time to death across other causes of death (**b**).

Indicators of the intensity of care in the last 12 months of life are shown in Table 2. Overall, 90.2% of cancer decedents were admitted to the hospital at least once in the last 12 months of life. Approximately 50% experienced inpatient care in the last 30 days of life, with 37.6% admitted multiple times during this period. Figure 1 reports average costs and healthcare utilisation across all cancers in the last year of life. Figure 2 shows average costs and healthcare utilisation by cancer types in the last year of life and reveals hospital inpatient and outpatient care as major drivers of costs. Figure 3 shows costs in the last 12 months of life for cancer versus other diseases. Cancer deaths had lower costs in the last month of life. However, resource use was higher in the last 90 days of life compared to other causes.

**Table 2:** Measures of care intensity in the last year of life, 2010–2017.

All cancers (*N* = 26,077)	*N*	%
Proportion admitted to hospital in the last year of life	23,522	90.2
Proportion admitted to hospital in the last 90 days of life	19,883	76.2
Proportion admitted to hospital in the last 30 days of life	12,883	49.4
Proportion >1 hospitalisation in the last month of life	9815	37.6
Proportion admitted emergency room visit in the last year of life	19,849	76.1
Proportion admitted emergency room visit in the 90 days last month of life	15,602	59.8
Proportion admitted emergency room visit in the 30 days of life	9808	37.6
Proportion >1 emergency room visit in the last 30 days of life	6255	23.9
Proportion admitted to the ICU in the last year of life	1561	6.0
Proportion admitted to the ICU in the 90 days of life	916	3.5
Proportion admitted to the ICU in the 30 days of life month of life	621	2.4

*ICU* = intensive care unit.

Among patients admitted at least once in the last year of life, the mean number of hospital admissions (for all cancer types) in the last year of life was 3.7 (SD, 5.8) and inpatient hospital length of stay was 25.3 days (SD, 27.7). The number of hospital admissions (mean, 7.2 [SD, 10.8]) and the total number of days spent in hospital (mean, 36.7 [SD, 33.0]) in the last year of life were highest for patients with haematological cancers. The proportion of decedents admitted to emergency rooms was high, with 76.1% presenting to the emergency room in the last 12 months of life and 37.6% in the last 30 days of life. The mean number of emergency visits in the last year of life was 1.7 (SD, 1.6) and was highest in prostate cancer decedents (mean 2.0 [SD, 1.9]). These patients were slightly older compared to other cancer groups. Detailed information on healthcare use in the last year of life is provided in Supplementary Appendix 2 and 3.

Around 6% of patients experienced at least one intensive care unit admission, with a mean length of stay of 5.7 days (SD, 7.3). Lung cancer decedents had the longest ICU length of stay (mean, 6.7 days [SD, 10.8]) and prostate cancer decedents had the lowest (mean, 4.6 days [SD, 4.4]). The overall mean number of outpatient attendances among all cancer decedents in the last year of life was 9.2 (SD, 14.2). Outpatient visits were highest for those with haematological cancers (mean 12.0, [SD, 14.4]) and lowest among decedents with unspecified primary site cancer (mean 6.5, [SD, 9.9]) and well as pancreatic cancer (mean 7.2 [SD, 9.8]).

The mean number of GP contacts in the last year of life was 26.8 (SD, 18.8) and was highest for the prostate cancer group (mean 30.8 [SD, 20.7]). Those who died of prostate cancer also had a higher number of telephone consultations, (mean 3.4 [SD, 5.4]), home visits (mean 2.6 [SD, 4.9]) and out-of-hours contacts (mean 1.3 [SD, 2.6]) in the last year of life. Breast cancer decedents had the highest mean of prescriptions (mean, 84.7 [SD, 92.2]), while people with haematological cancers had the lowest (mean, 66.5 [SD, 77.1]).

**Fig. 4:**
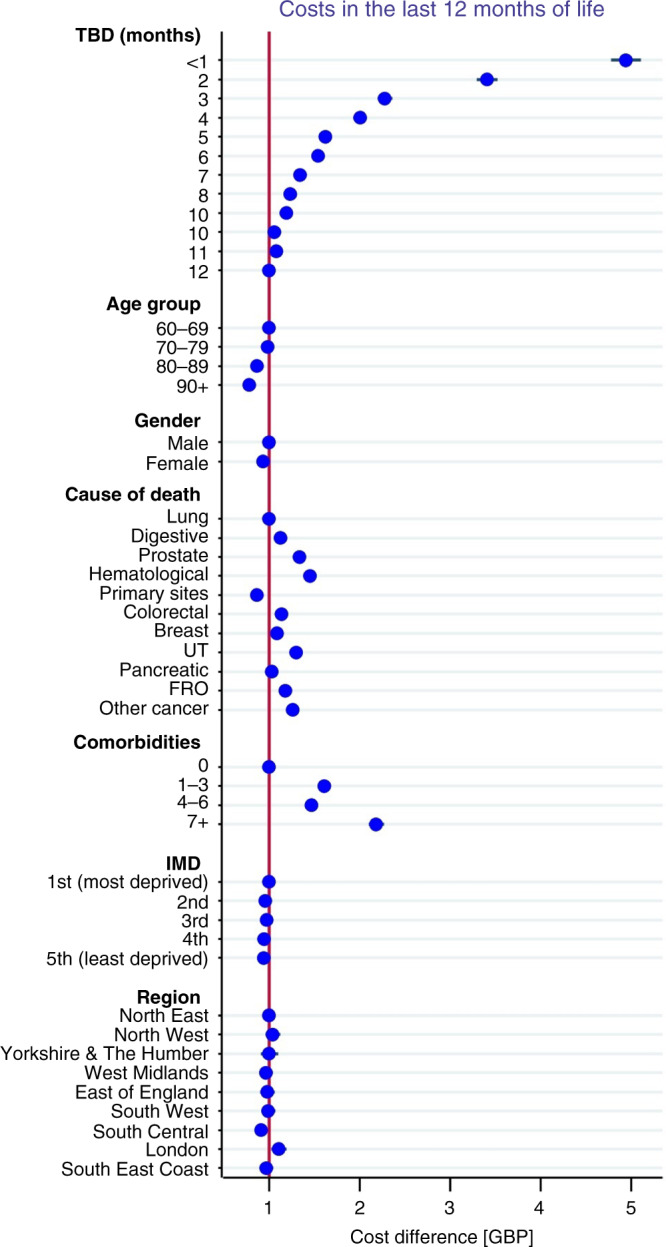
General linear model: healthcare costs in the last 12 months of life. Adjusted healthcare costs in the last 12 months of life across time to death (TBD), age, gender, comorbidity burden, cause of death, region of residence and Index of Multiple Deprivation (IMD).

Our models (Fig. 4) showed that cancer patients had highest costs in the last month of life and that hospital costs were the strongest driver of costs at the end-of-life in cancer patients. Costs decreased with age (0.78, 95% CI: 0.73–0.72, *p* < 0.005 for age group 90+ compared to the reference category age group 65–69). Costs increased substantially with higher comorbidity burden (2.2, 95% CI: 2.09–2.26, *p* < 0.005 for those with 7 or more comorbidities compared to those with 1–3 comorbidities) and were lower among females (0.93, 95% CI: 0.91–0.95, *p* < 0.000 compared to males). Costs were highest for patients with haematological cancers (1.45, 95% CI: 1.38–1.52, *p* < 0.005), urinary tract (1.30, 95% CI: 1.24–1.52, *p* < 0.005), and prostate cancer (1.28. 95% CI: 1.08–1.16, *p* < 0.005) patients, compared to the reference category (lung cancer). Costs were also higher for decedents in London compared to other regions (1.10, 95% CI: 1.02–1.19, *p* < 0.005).

Our analysis demonstrated that people who died from cancer had significantly higher rates of hospitalisation in the last month of life (1.28, 95% CI: 1.25–1.31, *p* < 0.005) than over the preceding months. Those who died from haematological cancers had higher rates of hospital admissions (0.91, 95% CI: 0.88–0.93, *p* < 0.005) compared to those with other cancers. Women were less likely to be admitted to hospital in the last year of life (−0.12, 95% CI: −0.14 to −0.11, *p* < 0.000). Individuals with the greatest comorbidity burden (7+ comorbidities) had significantly higher rates of hospitalisation in the last year of life (1.28, 95% CI: 1.26–1.31, *p* < 0.000) than those with a lower comorbidity burden.

## Discussion

### Main findings

This study expands the scope of existing population-based, patient-level data to include an analysis of the costs of primary and secondary healthcare accessed by patients with cancer in England during the last year of life. Healthcare utilisation and costs increased sharply in the last month of life; most notably hospitalisation frequency and associated costs. Hospital costs were by far the largest cost element. The hospitalisation was most common in younger patients, males, individuals with haematological cancers, those with higher comorbidity burden the least socioeconomically deprived and those living in the London region. Our findings revealed a high frequency of primary care use overall with the highest primary care users being those with prostate cancer.

### Comparison with previous research

Our findings are consistent with previous research in England showing that people with cancer were the highest users of healthcare resources use at the end-of-life. A 2017 [22] study of cancer patients, using data from multiple national databases in England, found that hospital costs for cancer patients increased substantially at the end-of-life and that patients with lower socioeconomic status had higher costs compared with patients with higher socioeconomic status. Another study [23] analysed healthcare use by people with urological cancers in England over several years. The authors found a sharp increase in secondary care activity in the last year of life, consistent with our own findings, though also identified increasing outpatient attendances. In our study, outpatient attendances remained relatively stable and increased only gradually over the last year of life, with the largest change observed in the last two months of life. Gao et al. [24] investigated factors associated with General Practitioner consultation by cancer patients in the last year of life, also using CPRD data. In line with our findings, the authors found that younger age, higher comorbidity burden and prostate cancer were associated with more primary care consultations. Others’ studies based in the United Kingdom also reported increased costs in cancer patients [1, 25, 26]. Our findings are in line with previous research from North America, which showed that end-of-life care intensity declined with advancing age [27, 28].

High rates of hospital admissions and inpatient bed days near the end-of-life among cancer patients have been reported in many different countries including Belgium, Canada, England, Germany, the Netherlands, Norway, and the United States and Australia [4, 12, 29–32].

### Implications of our findings for practice and further research

There are many reasons why people with advanced cancer who are nearing the end-of-life may require inpatient hospitalisation. We understand that as cancer advances, individuals typically experience escalating symptoms, deteriorating physical function, and associated increasing care needs. One hypothesis is that acute hospital care represents the most accessible and responsive care offered when people’s needs change, even when their preferences may be for home-based care. Inpatient palliative care interventions have been shown to be cost-saving and beneficial to the overall patient care [33, 34].

The observed variation in hospital use towards end-of-life between patients with different cancer types may reflect their varied cancer treatment pathways. It is understood that people with certain cancer types are more likely to still be receiving clinical interventions late in their disease, with anti-cancer treatments such as blood product transfusions for people with haematological being one example (potentially accounting for some of their high-intensity acute hospital use towards the end-of-life). Furthermore, some of the variations in healthcare use by cancer type may also be accounted for by the length of time that individuals lived with their cancer. For example, patients who presented with advanced disease at diagnosis, and who lived for only weeks or short months, will likely have had different treatment and care trajectories, when compared with patients who lived with their cancer for longer. Our findings revealed lower hospitalisation rates for older patients. There is some evidence that younger patients are more likely to receive more aggressive and costly treatment modalities, including intensive care, surgery and chemotherapy, which might in turn explain the increased costs observed [1]. Recent oncological innovations in imaging, radiation therapy approaches and drug development have come at a considerable increase in costs, any of which may be higher in younger populations if they are more likely to receive treatment [35]. We found evidence that people with haematological cancers experienced particularly high rates of hospitalisation and associated costs when compared with care for those with other cancer types. It is recognised that patients with haematological cancers often receive inpatient medical interventions until close to death, with the example of blood transfusion already highlighted, and with a significant proportion of patients dying in hospital [36]. Others’ research has documented lower rates of hospice use and higher rates of chemotherapy among patients with hematologic malignancies [7, 37].

There is growing interest in developing more integrated services between haematology and specialist palliative care to support advanced care planning in the hope that this will improve shared decision making and reduce undesirable hospital care at end-of-life [38, 39]. It is increasingly understood that people with haematological cancers may have unmet palliative care needs and that there can be uncertainty about the optimal timing of palliative care in the patient journey [40]. It has been suggested that an understanding of the unique trajectories of people with haematological cancers is needed by specialist palliative care teams [41] and also that early, integrated palliative care alongside haematology treatments can be beneficial, with improved physical and psychological symptom control amongst other benefits [42]. A collaborative approach, both within multidisciplinary haematology teams, but also between haematology and specialist palliative care teams, has been proposed [43]. Our data support the ongoing need for inpatient specialist palliative care services in both acute and elective oncology settings with such a high proportion of cancer patients spending significant amounts of time in hospital in the last year and months of life. We have previously demonstrated that most evidence on the cost-effectiveness of palliative and end-of-life care interventions is related to home-based interventions, with associated substantial reductions in total healthcare costs, resource use and improvement in patient outcomes [44]. Considering these new data in light of this study, our data provide support for investment in home-based palliative and end-of-life care, which together with appropriate social care, might offer dual benefits of improving outcomes and experiences for patients, and at reduced system costs.

However, it is also the case that we do not understand enough about the details of why people with advanced cancer are admitted to hospitals and the extent to which inpatient care is actually meeting their needs. Until we know more about the factors that contribute to admission, and what happens during admissions near the end-of-life, we cannot draw conclusions about whether care needs and preferences could be better met in alternative settings.

### Strengths and limitations

Our study has several strengths and limitations. This is the largest study of costs and service use at the end-of-life in cancer patients in England between 2010 and 2017. The greatest strength of this study is the multiple dimensions of healthcare data. This allowed tracking patients interactions with primary and secondary care, planned and unscheduled. The findings of this study provide important insights into the cost burden and intensity of care across different types of cancer types. There is a need for large representative samples of cancer patients to determine the intensity of care and costs associated with care provided to cancer patients near death. Our sample included over 26,000 cancer patients aged 65 and older. Cancer remains the second leading cause of death in both the UK and the US. In our study, we focus on cancer patients in England who are 65 and older, enabling direct comparison with other US studies of cancer patients who are Medicare recipients. Moreover, given potential financial incentives to overtreat dying cancer patients in the US, our study provides an important comparison with care intensity and costs in the UK healthcare system; where the overwhelming majority of cancer and end-of-life care is delivered free of charge to individuals and free from insurance-related billing.

Our study has a number of limitations which are mainly related to due to nature of secondary data sources used in the analysis. We used CPRD rather than the whole population to analyse contacts with primary care and northern regions were not well represented compared to southern regions. Also, we were not able to include information on the use and costs of social and informal care and therefore we underestimate the economic burden. Previous studies have suggested that costs of social care represent only a small proportion of total costs [1, 45]. In our analysis, we neither report the cancer stage nor the date of diagnosis. In this study we were unable to identify routine health data which demonstrated the receipt of palliative care in our study population. Firstly, the majority of palliative care in the UK is integrated as part of the care delivered by primary and secondary healthcare teams (e.g. oncology clinicians, general practitioners and district nurses). As such, it is not reliably coded as palliative care. Secondly, Specialist Palliative Care, delivered by dedicated teams, is commonly part-charitably funded, with clinical data sitting outside NHS datasets. We considered various proxies for palliative care receipt but found major inconsistencies that rendered these impossible for inclusion in our study. The incorporation of robust direct measures of the provision and impact of palliative care for patients with cancer would add value to future studies in this area.

The data did also not permit analysis of anticipatory care planning/other measures of palliative care. Also, qualitative data relating to patient/family experiences of care and the extent to which they felt their care and support needs were met was not available. Finally, our data are from 2010 to 2017 and therefore do not cover the period of the SARS-CoV-2 (COVID-19) pandemic, which significantly disrupted healthcare delivery and led to unprecedented pressures on critical care beds [46]. The risk of developing severe COVID-19 illness has been shown to be particularly high in cancer patients [47–49] and COVID-19-related mortality for people with cancer has been shown to be as high as 20–30% [50, 51]. Most COVID-19-related deaths have occurred in those over 65 s [52] and there has been major recognition of the need for widespread advanced care planning [53–55].

## Conclusions

Our study has revealed escalating healthcare use and costs over the last year of life in a large population with advanced cancer in England. The intensity of healthcare use and associated costs were particularly high during the last month of life, and most markedly so for those with haematological cancers and wider demographic characteristics including younger age. Further research is needed to understand more about distinct cancer populations’ pathways and experiences before recommendations can be made about the most appropriate models of care for people with advanced cancer who are nearing the end-of-life.

## Supplementary information

Supplementary Information file

Checklist

## Data Availability

The data are not publicly available due to privacy and ethical restrictions.
