# Supplementary material for: Intensity of care in cancer patients in the last year of life: a retrospective data linkage study
Source: Br J Cancer. 2022 May 11;127(4):712–9. doi: 10.1038/s41416-022-01828-0 (PMC9092325; doi:10.1038/s41416-022-01828-0)
Supplement: Supplementary file 1 — Supplementary Information file [file 41416_2022_1828_MOESM1_ESM.pdf]

Supplementary Appendix 1. Patients' characteristics across cancer sites, 2010–2017

Characteristics	Lung	Digestive	Prostate	Haematological	Primary sites	Colorectal	Breast	Urinary tract	Pancreatic	FRO	Other	Total
**n, %**												
**Gender**												
Men	2, 808 (54.36)	2,524 (57.89)	2,197 (100.00)	1,127 (53.85)	805 (46.26)	858 (49.48)	11 (0.66)	1,016 (62.52)	634 (45.84)	0 (0.00)	1,739 (58.22)	13,719 (52.61)
Women	2,358 (45.64)	1,836 (42.11)	0 (0.00)	966 (46.15)	935 (53.74)	876 (50.52)	1,652 (99.34)	609 (37.48)	749 (54.16)	1,129 (100.00)	1,248 (41.78)	12,358 (47.39)
**Age**												
65-69	436 (8.44)	233 (5.34)	66 (3.0)	114 (5.45)	111 (6.38)	80 (4.61)	97 (5.83)	89 (5,48)	92 (6.65)	86 (7.62)	209 (7.0)	1,613 (6.19)
70-79	2,347 (45.4)	1,680 (38.53)	681 (31.0)	747 (35.69)	587 (33.74)	564 (32.53)	563 (33.85)	511 (31.45)	588 (42.52)	475 (42.07)	1,150 (38.50)	9,893 (37.94)
80-89	1,933 (37.42)	1,836 (42.11)	1,017 (46.29)	939 (44.86)	730 (41.95)	779 (44.93)	638 (38.36)	728 (44.80)	548 (39.62)	423 (37.47)	1,196 (40.04)	10, 767 (41.29)
90+	450 (8.71)	611 (14.01)	433 (19.71)	293 (14.0)	312 (17.94)	311 (17.94)	365 (21.95)	297 (18.28)	155 (11.21)	145 (12.84)	432 (14.46)	3,804 (14.59)
**Comorbidities**												
0	1,231 (23.83)	829 (19.01)	823 (37.46)	415 (19.83)	504 (28.97)	447 (25.78)	635 (38.18)	404 (24.86)	309 (22.34)	280 (24.80)	1032 (34.55)	6,909 (26.49)
1-3	3,076 (59.54)	2,950 (67.66)	1,000 (45.52)	1,565 (74.77)	396 (22.76)	982 (56.63)	520 (31.27)	987 (60.74)	908 (65.65)	562 (49.78)	1509 (50.52)	14, 445 (55.43)
4-6	316 (6.12)	269 (6.17)	145 (6.60)	67 (3.20)	637 (36.61)	118 (6.81)	242 (14.55)	95 (5.85)	74 (5.35)	88 (49.78)	210 (7.03)	2,261 (8.67)
7+	543 (10.51)	312 (7.16)	229 (10.42)	46 (2.20)	203 (11.67)	187 (10.78)	266 (16.00)	139 (8.55)	92 (6.65)	199 (17.63)	236 (7.90)	2,452 (9.40)
**IMD[a]**												
1st (most deprived)	997 (19.30)	953 (21.86)	570 (25.94)	550 (26.28)	392 (22.53)	435 (25.09)	388 (23.33)	392 (24.12)	350 (25.31)	265 (23.47)	747 (25.01)	6,039 (23.16)
2nd	993 (19.22)	972 (22.29)	532 (24.21)	492 (23.51)	388 (22.30)	379 (21.86)	394 (23.69)	372 (22.89)	309 (22.34)	259 (22.94)	716 (23.97)	5,806 (22.26)
3rd	1,050 (20.33)	955 (21.90)	494 (22.49)	492 (23.51)	380 (21.84)	368 (21.22)	388 (23.33)	356 (21.91)	341 (24.66)	248 (21.97)	668 (22.36)	5,740 (22.01)
4th	1,090 (21.10)	845 (19.38)	365 (16.61)	317 (15.5)	308 (17.70)	301 (17.36)	272 (16.36)	289 (17.78)	225 (16.27)	203 (17.98)	510 (17.07)	4,725 (18.12)
5th (least deprived)	1,036 (20.05)	635 (14.56)	236 (10.74)	242 (11.56)	272 (15.63)	251 (14.48)	221 (13.29)	216 (13.29)	158 (11.42)	154 (13.64)	346 (11.58)	3,767 (14.45)
**Region**												
North East	109 (2.11)	57 (1.31)	33 (1.5)	33 (1.58)	25 (1.44)	25 (1.44)	23 (1.38)	27 (1.66)	25 (1.81)	11 (0.97)	52 (1.74)	420 (1.61)
North West	1, 040 (20.13)	715 (16.40)	275 (12.52)	273 (13.04)	278 (15.98)	255 (14.71)	215 (12.93)	254 (15.63)	173 (12.51)	138 (12.22)	421 (14.09)	4,037 (15.48)
Yorkshire & The Humber	137 (2.65)	103 (2.36)	47 (2.14)	53 82.53)	43 (2.47)	36 (2.08)	33 (1.98)	29 (1.78)	18 (1.30)	30 (2.66)	49 (1.64)	578 (2.22)
West Midlands	607 (11.75)	621 (14.24)	300 (13.65)	274 (13.09)	228 (13.10)	224 (12.92)	200 (12.03)	207 (12.74)	175 (12.65)	162 (14.35)	361 (12.09)	3,359 (12.88)
East of England	391 (7.57)	338 (7.75)	182 (8.28)	181 (8.65)	147 (8.45)	147 (8.48)	167 (10.04)	134 (8.25)	109 (7.88)	80 (7.09)	296 (9.91)	2,172 (8.33)
South West	561 (10.86)	484 (11.10)	295 (13.43)	252 (12.04)	201 (11.55)	224 (12.92)	209 (12.57)	201 (12.37)	178 (12.87)	138 (12.22)	364 (12.19)	3,107 (11.91)
South Central	767 (14.85)	686 (15.73)	362 (16.48)	352 (16.82)	287 (16.49)	279 (16.09)	271 (16.30)	274 (16.86)	237 (17.14)	205 (18.16)	504 (16.87)	4,224 (16.20)
London	583 (11.29)	485 (11.12)	239 (10.88)	244 (11.66)	207 (11.90)	173 (9.98)	175 (10.52)	148 (9.11)	159 (11.50)	139 (12.31)	330 (11.05)	2,882 (11.05)
South East Coast	971 (18.80)	871 (19.98)	464 (21.12)	431 (20.59)	324 (18.62)	371 (21.40)	370 (22.25)	351 (21.60)	309 (22.34)	226 (20.02)	610 (20.42)	5,298 (20.32)
Total	5,166 (19.81)	4,360 (16.72)	2,197 (8.43)	2,093 (8.03)	1,740 (6.67)	1,734 (6.65)	1,663 (6.38)	1,625 (6.23)	1,383 (5.30)	1,129 (100.00)	2, 987 (100.00)	26,077 (100.00)

FRO = female reproductive organs; **IMD** = Index of Multiple Deprivation

Supplementary Appendix 2. Health Care Utilization in the Last Year of Life for cancer decedents (2010-2017)

**Health care utilisation mean (SD)**	**All cancers**	**Lung**	**Digestive**	**Prostate**	**Haematological**	**Primary site**	**Colorectal**	**Breast**	**UT**	**Pancreatic**	**FRO**	**Other cancers**
**Last 12 months of life**												
Inpatient care												
Hospital admissions	3.7 (5.8)	3.1 (4.1)	3.7 (4.5)	3.4 (4.7)	7.2 (10.8)	2.4 (4.4)	3.5 (4.6)	3.4 (5.0)	3.8 (7.5)	3.6 (4.7)	4.4 (5.4)	3.7 (6.4)
LOS (total in days)	25.3 (27.7)	21.0 (26.2)	25.3 (27.3)	26.6 (28.9)	36.7 (33.0)	21.3 (23.0)	23.9 (25.9)	20.7 (23.7)	31.5 (30.9)	22.2 (21.7)	26.8 (26.0)	27.3 (29.2)
Intensive care (ICU)												
ICU admissions	1.0 (0.2)	1.0 (0.2)	1.0 (0.2)	1.0 (0.2)	1.0 (0.1)	1.0 (0.3)	1.0 (0.2)	1.0 (0.2)	1.0 (0.1)	1.0 (0.1)	1 (0)	1.0 (0.2)
ICU LOS	5.7 (7.3)	6.7 (9.9)	5.9 (7.1)	4.6 (4.4)	5.7 (5.1)	6.5 (10.7)	5.3 (5.4)	4.8 (4.7)	5.7 (7.0)	5.5 (5.0)	5.3 (12.4)	5.2 (6.6)
Emergency room												
ER visits	1.7 (1.6)	1.7 (1.7)	1.6 (1.6)	2.0 (1.9)	1.8 (1.7)	1.4 (1.4)	1.5 (1.5)	1.4 (1.5)	1.9 (1.8)	1.5 (1.5)	1.6 (1.5)	1.7 (1.7)
Outpatient care												
Outpatient attendances	9.1 (14.2)	9.2 (17.3)	8.5 (12.6)	9.3 (13.4)	12.0 (14.4)	6.5 (13.8)	8.5 (15.9)	9.2 (12.3)	8.9 (11.8)	7.2 (9.8)	10.1 (13.9)	10.6 (13.5)
**Last 90 days of life**												
Inpatient care												
Hospital admissions	1.5 (1.9)	1.5 (1.8)	1.5 (1.6)	1.3 (1.4)	2.8 (3.4)	1.3 (1.3)	1.3 (1.5)	1.2 (1.5)	1.5 (2.2)	1.6 (1.6)	1.5 (1.6)	1.5 (2.0)
LOS (total in days)	14.2 (16.3)	13.4 (16.7)	13.6 (15.7)	13.5 (16.3)	20.1 (18.3)	14.5 (15.1)	12.0 (14.9)	11.2 (14.9)	16.5 (17.4)	13.2 (13.8)	14.8 (15.9)	14.4 (16.8)
Intensive care (ICU)												
ICU admissions	1.0 (0.2)	1.0 (0.2)	1.1 (0.2)	1.0 (0.2)	1.0 (0.1)	1.0 (0.3)	1.0 (0.3)	1.0 (0.2)	1.0 (1.1)	1.0 (1.2)	1.0 (0)	1.0 (0.2)
ICU LOS	6.1 (7.0)	7.1 (9.0)	6.1 (7.6)	5.3 (4.7)	5.9 (5.4)	6.9 (12.5)	6.1 (6.4)	5.6 (5.4)	6.1 (6.3)	6.3 (6.0)	3.8 (2.5)	5.8 (6.5)
Emergency room												
ER visits	0.9 (0.9)	0.9 (1.0)	0.8 (0.9)	0.9 (1.0)	1.0 (1.0)	0.9 (0.9)	0.7 (0.9)	0.7 (0.9)	0.9 (1.0)	0.8 (0.9)	0.8 (0.8)	0.8 (0.9)
Outpatient care												
Outpatient attendances	2.8 (5.8)	3.2 (6.4)	2.6 (6.0)	2.6 (5.3)	3.7 (5.6)	2.3 (5.6)	2.4 (6.9)	2.7 (5.2)	2.8 (5.2)	2.3 (3.9)	2.7 (5.1)	3.1 (5.8)
**Last 30 days of life**												
Inpatient care												
Hospital admissions	0.6 (0.9)	0.7 (1.2)	0.6 (0.8)	0.5 (0.7)	1.0 (1.3)	0.7 (0.7)	0.5 (0.7)	0.5 (0.7)	0.5 (0.8)	0.6 (0.9)	0.5 (0.7)	0.6 (0.8)

LOS (total in days)	5.4 (8.7)	5.8 (11.6)	5.1 (7.5)	4.5 (7.2)	7.6 (9.7)	6.6 (8.2)	4.5 (7.3)	4.1 (7.0)	5.4 (7.9)	5.4 (7.4)	5.2 (7.9)	4.9 (7.5)
Intensive care (ICU)												
ICU admissions	1.0 (0.2)	1.0 (0.3)	1.0 (0.3)	1.0 (0.3)	1.0 (0.1)	1.0 (0.3)	1.0 (0.2)	1.0 (0.0)	1.0 (0.2)	1.0 (0.2)	1.0 (0.0)	1.0 (0.2)
ICU LOS	5.8 (5.0)	7.0 (6.3)	5.9 (5.3)	4.9 (5.6)	5.6 (4.2)	5.3 (4.0)	5.2 (4.0)	5.9 (6.2)	4.7 (3.0)	6.7 (6.7)	3.2 (1.4)	6.0 (5.1)
Emergency room												
ER visits	0.4 (0.6)	0.5 (0.6)	0.4 (0.6)	0.4 (0.6)	0.5 (0.6)	0.5 (0.6)	0.3 (0.6)	0.3 (0.5)	0.4 (0.6)	0.4 (0.6)	0.3 (0.5)	0.4 (0.6)
Outpatient care												
Outpatient attendances	0.9 (0.3)	1.1 (3.7)	0.8 (3.4)	0.8 (2.8)	1.1 (2.8)	0.8 (2.3)	0.8 (3.4)	0.8 (2.3)	0.9 (3.0)	0.7 (2.2)	0.8 (2.9)	0.8 (3.0)

Abbreviations: UT = urinary tract; FRO = female reproductive organs; ICU = Intensive Care Unit, LOS = length of stay; ER: emergency room

Supplementary Appendix 3. Primary Care Utilization in the Last Year of Life for cancer decedents (2010-2017)

Primary care contacts, mean (SD)	All cancers	Lung	Digestive	Prostate	Haematological	Primary site	Colorectal	Breast	Urinary tract	Pancreatic	FRO	Other cancers
**Last 12 months of life**												
GP consultations	26.8 (18.8)	26.2 (17.8)	26.6 (18.3)	30.8 (20.7)	27.8 (19.4)	22.6 (17.0)	26.1 (19.0)	25.0 (18.5)	28.0 (19.5)	26.9 (18.5)	27.8 (18.7)	27.5 (18.9)
Telephone consultations	2.8 (4.6)	2.6 (4.5)	2.8 (4.9)	3.4 (5.4)	2.5 (4.4)	2.5 (4.2)	2.8 (4.7)	2.6 (4.2)	3.3 (5.1)	2.9 (4.5)	3.2 (5.0)	2.6 (4.4)
Home visits	1.9 (4.5)	1.8 (5.1)	1.9 (4.0)	2.6 (4.9)	1.5 (3.4)	1.6 (3.2)	2.2 (5.4)	2.4 (4.8)	2.2 (4.8)	1.5 (2.8)	2.1 (3.9)	2.0 (4.7)
Out-of-hours	0.8 (1.9)	0.8 (1.6)	0.8 (1.7)	1.3 (2.6)	0.7 (1.5)	0.7 (1.6)	0.9 (1.9)	0.8 (1.8)	1.2 (2.2)	0.9 (1.6)	1.0 (1.9)	0.8 (1.7)
Medications	76.4 (81.9)	78.1 (81.4)	76.4 (86.2)	86.3 (83.9)	66.5 (77.1)	70.6 (77.0)	75.7 (82.6)	84.7 (92.2)	76.9 (81.3)	73.9 (81.9)	74.7 (73.6)	73.9 (78.8)
**Last 90 days of life**												
GP consultations	10.3 (7.5)	10.7 (7.4)	10.4 (7.7)	10.4 (7.6)	9.5 (7.0)	9.7 (7.3)	9.9 (7.5)	9.3 (7.1)	10.3 (7.8)	11.4 (7.7)	10.5 (7.6)	10.3 (7.6)
Telephone consultations	1.3 (2.3)	1.2 (2.1)	1.3 (2.2)	1.4 (2.5)	1.1 (2.1)	1.3 (2.3)	1.3 (2.4)	1.1 (2.2)	1.4 (2.5)	1.5 (2.4)	1.4 (2.4)	1.2 (2.2)
Home visits	1.2 (2.4)	1.0 (2.1)	1.2 (2.3)	1.4 (2.8)	0.8 (1.9)	1.1 (2.1)	1.2 (2.4)	1.2 (2.5)	1.2 (2.7)	1.0 (2.0)	1.3 (2.2)	1.2 (2.7)
Out-of-hours	0.6 (1.2)	0.5 (1.2)	0.5 (1.2)	0.7 (1.6)	0.4 (0.9)	0.5 (1.0)	0.6 (1.2)	0.5 (1.2)	0.7 (1.5)	0.6 (1.3)	0.6 (1.3)	0.5 (1.1)
Medications	24.1 (25.6)	25.8 (26.2)	23.8 (25.7)	26.3 (26.4)	18.9 (24.3)	22.2 (23.8)	23.9 (24.7)	25.8 (27.5)	23.8 (25.7)	24.7 (25.1)	24.0 (23.7)	23.7 (25.2)
**Last 30 days of life**												
GP consultations	4.3 (3.6)	4.5 (3.5)	4.3 (3.5)	4.1 (3.6)	3.8 (3.4)	4.3 (3.6)	4.2 (3.6)	3.8 (3.4)	4.2 (3.7)	4.9 (3.7)	4.3 (3.6)	4.1 (3.6)
Telephone consultations	0.5 (1.1)	0.5 (1.1)	0.5 (1.1)	0.5 (1.1)	0.4 (1.1)	0.6 (1.2)	0.6 (1.2)	0.5 (1.1)	0.5 (1.2)	0.6 (1.3)	0.6 (1.2)	0.5 (1.1)
Home visits	0.6 (1.3)	0.6 (1.2)	0.6 (1.3)	0.7 (1.5)	0.4 (1.1)	0.6 (1.4)	0.7 (1.5)	0.6 (1.4)	0.6 (1.5)	0.6 (1.3)	0.7 (1.3)	0.6 (1.4)
Out-of-hours	0.3 0.8	0.3 (0.7)	0.3 (0.8)	0.4 (0.9)	0.3 (0.7)	0.3 (0.8)	0.4 (0.9)	0.3 (0.8)	0.4 (0.9)	0.4 (0.8)	0.4 (0.9)	0.3 (0.8)
Medications	8.9 (10.9)	9.7 (11.3)	8.9 (11.0)	9.5 (11.5)	6.7 (10.2)	8.0 (9.9)	9.3 (11.0)	9.3 (11.3)	8.7 (11.9)	9.7 (11.7)	9.1 (10.7)	8.9 (10.9)

Abbreviations: FRO = female reproductive organs
